# The Geogenomic Mutational Atlas of Pathogens (GoMAP) Web System

**DOI:** 10.1371/journal.pone.0092877

**Published:** 2014-03-27

**Authors:** David P. Sargeant, Michael W. Hedden, Sandeep Deverasetty, Christy L. Strong, Izua J. Alaniz, Alexandria N. Bartlett, Nicholas R. Brandon, Steven B. Brooks, Frederick A. Brown, Flaviona Bufi, Monika Chakarova, Roxanne P. David, Karlyn M. Dobritch, Horacio P. Guerra, Kelvy S. Levit, Kiran R. Mathew, Ray Matti, Dorothea Q. Maza, Sabyasachy Mistry, Nemanja Novakovic, Austin Pomerantz, Timothy F. Rafalski, Viraj Rathnayake, Noura Rezapour, Christian A. Ross, Steve G. Schooler, Sarah Songao, Sean L. Tuggle, Helen J. Wing, Sandy Yousif, Martin R. Schiller

**Affiliations:** School of Life Sciences, University of Nevada Las Vegas, Las Vegas, Nevada, United States of America; University of Otago, New Zealand

## Abstract

We present a new approach for pathogen surveillance we call Geogenomics. Geogenomics examines the geographic distribution of the genomes of pathogens, with a particular emphasis on those mutations that give rise to drug resistance. We engineered a new web system called Geogenomic Mutational Atlas of Pathogens (GoMAP) that enables investigation of the global distribution of individual drug resistance mutations. As a test case we examined mutations associated with HIV resistance to FDA-approved antiretroviral drugs. GoMAP-HIV makes use of existing public drug resistance and HIV protein sequence data to examine the distribution of 872 drug resistance mutations in ∼502,000 sequences for many countries in the world. We also implemented a broadened classification scheme for HIV drug resistance mutations. Several patterns for geographic distributions of resistance mutations were identified by visual mining using this web tool. GoMAP-HIV is an open access web application available at http://www.bio-toolkit.com/GoMap/project/

## Introduction

There are a number of computation tools for visualizing pathogen surveillance data on world atlases. An example is the NCHHSTP Atlas web application at the United States Center for Disease Control and Prevention (CDC; www.cdc.gov). This, and similar applications are useful in managing responses to pathogen outbreaks. There are several examples of pathogens that have acquired mutations that make them resistant to one or more drugs. Indeed, this is a serious concern for treatment of patients infected with *Staphylococcus aureus*, *Clostridium difficile, Streptococcus pyogenes, Mycobacterium tuberculosis*, *Salmonella*, *Escherichia coli*, Influenza viruses, Human Immunodeficiency Virus (HIV), and several other viral infections. As sequencing costs plummet, pathogen genotyping is likely to become a routine diagnostic tool. Thus, examining the geographic distribution of mutations that are associated with drug resistance has the potential to contribute to the management of pathogen treatments. We call this approach Geogenomics.

To demonstrate the usefulness and feasibility of Geogenomics, we built a new computational system called Geogenomic Mutational Atlas of Pathogens (GoMAP). Since our first application is to HIV, the version presented herein is called GoMAP-HIV. We chose HIV for several reasons. Approximately 40 million people are infected with HIV worldwide, presenting a daunting health care challenge [1] for which there are over 25 drug compounds approved by the Food and Drug Administration (FDA) [2]. Although Highly Active Antiretroviral Therapy (HAART) provides an effective approach to treating HIV infection, there is significant *de novo* and transmission based drug resistance, especially if the treatment regimen is followed imperfectly [3]–[5]. For example, the rate of drug resistance in drug-naïve populations of Africa, East and Southeast Asia, North America, and Europe ranges from 5–12% [6].

Several databases (Stanford Database, World Health Organization (WHO), and Los Alamos) have consolidated many of the currently known HIV drug resistance mutations (DRMs). Another source of HIV data is the HIV genome sequences from many different patient blood samples housed in a number of databases. The largest of these is the Los Alamos HIV sequence database, which contains the genomic sequences from different patient blood samples encoding ∼502,000 sequences of HIV proteins. While these two large datasets each provide a valuable resource, both can be used together to identify the occurrence of individual mutations in individual countries; however, this has not yet been reported.

The presence of HIV drug resistance mutations in the human population can be geographically assigned and the results may be useful for tracking the spread of DRMs, educating about HIV drug resistance, advancing our understanding of HIV evolution, and potentially for the management and design of therapies. To this end, we present the GoMAP-HIV open-access web application, which provides a visual mining tool to help scientists better understand the global distribution of HIV DRMs.

## Results

### Data sources for the GoMAP-HIV database

Using the HIVToolbox MySQL database as a starting point, 1571 known HIV-1 DRMs (872 for FDA-approved drugs) were added from the Los Alamos and Stanford HIV databases, World Health Organization website, International AIDS Society and primary literature [7]–[9]. The HIVToolbox database has ∼502,000 total sequences from patient blood samples in 126 different countries [10]. The appearance of DRMs in a country was determined by performing multiple sequence alignments of protein sequences from patient blood samples with an HIV reference protein as previously created using ClustalΩ to generate clade specific alignments in the HIVToolbox database [11].

### GoMAP-HIV User Interface

The main interface is composed of two primary sections, the Naïve and Treated Atlas Window and the DRM Selection and Display Tools.

#### Naïve and Treated Atlas Window

**Figure 1:**
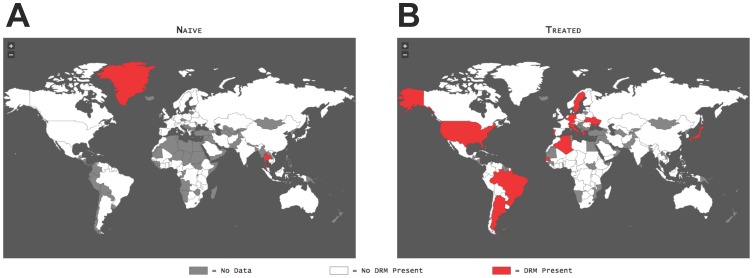
GoMAP example maps. World atlas displays showing observation of DRMs derived from patient blood samples for naïve (**A**) and treated (**B**) patients. The examples shown are for the beneficial N88S DRM for Fosamprenavir. A color legend for country coloring is shown (bottom).

This window consists of two panels, each displaying data for naïve or treated patients on an atlas of all recognized countries (**Fig. 1**). When a user first enters the GoMAP-HIV site, the countries in both atlases are initially white. Hovering the mouse over each country results in the appearance of a label containing the country name (**Fig. 1A**). The user can zoom in or out of each of the atlases using the plus and minus icons located in the top left corner of each panel.

The user can first select a country first or a drug. Upon selection of a country, antiretroviral (ARV) drug, and then a DRM from the selection windows, color-coded countries are displayed on the atlases (**Fig. 1**). The colors are: white indicates no observed DRM, red indicates at least one sequence containing the DRM, and gray indicates those countries that do not have sequences in our database. The left atlas panel is driven by patient blood sample sequence data obtained from treatment-naïve patients while treatment-experienced patient blood sample data drives the right panel (**Fig. 1A and 1B**, respectively). In order to populate the atlases with the DRM data, one must first choose from the DRM Selection Tools found below the atlas panels. The following are found under the DRM Selection Tools:

#### Country Selection window

**Figure 2:**
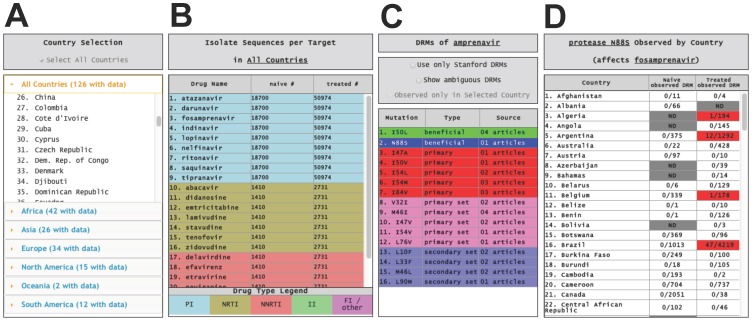
GoMAP country, drug, and DRM data selectors. **A.** The Country Selection window is used to load data into world atlas displays and populate the Drug Selection table. **B.** The Drug Selection window is for choosing an FDA-approved drug. ARVs are grouped by type: Protease inhibitors (PI) are colored a light blue, nucleoside/nucleotide reverse transcriptase inhibitors (NRTI) are tan, non-nucleoside reverse transcriptase inhibitors (NNRTI) are coral, integrase inhibitors (II) are light green, and fusion inhibitors (FI) are light magenta. The number of different patient blood samples for a country selected in A is shown. If the “Select All Countries” checkbox is checked, data for all countries are shown. Selection of a drug populates the DRM Selection table with DRM data for that drug, and country if selected. **C.** The DRM Selection table shows DRMs ordered by their classification. Once a DRM is selected (N88S here) the presence of DRMs in each country are plotted onto the world atlases and the DRM Observation By Country table (**D**) is populated. The DRM Selection table also has a hyperlink to one or more published papers supporting the DRM. Checkboxes below the title bar enable display of DRMs present in other databases for which no published scientific paper could be identified (designated ambiguous), for showing the Major/Minor DRM classification scheme, and for examining DRMs only in the country selected. **D.** The DRM Observation By Country table shows a list of the observed DRMs (numerator) and total sequences examined (denominator) for each country. The data are stratified for naïve and treated patients.

This selector consists of seven expandable categories organized by geographic region. When the user first enters the GoMAP-HIV site, all countries are selected as indicated by the checkbox in the tile bar (**Fig. 2A**). The “All Countries (126 with data)” category consists of an alphabetical list of the countries in the database that have associated DRMs. Thus far, we have HIV sequence data from 126 out of the 253 recognized countries of the world. The other six categories contain the same country information, but the countries are grouped by continent. To access DRM data for a specific country, the user selects that country by clicking on the country name found in either the “All Countries” or continent category, or by using the mouse to click on a country in the atlas displays. Once the country selection has been made, this updates information into the Drug Selection window. At any time, checking the “Select All Countries” checkbox will update data for all countries in the world.

#### Drug Selection window

Initially, this window contains a list of 23 FDA-approved drugs with the total number of sequences for the target of each drug. This window is updated after selection of a country. The title bar displays the name of the selected country. The Drug Selection window is populated with up to 23 FDA approved drugs that have sequence data for the HIV protein drug target (**Fig. 2B**). The FDA drugs are color-coded based on inhibitor type, with the color-code legend located at the bottom of the drug selection window. The first column of the table in this window contains the drug names. The second and third columns contain the number of naïve-patient sequences or treated-patient sequences for the country selected. Selection of a drug from the first column triggers two events: 1) indication of the drug selected in the DRM selection Title bar, and 2) population of the DRM Selection window with DRMs for the ARV selected.

#### DRM Selection window

Each row of the DRM selection table shows information about a DRM for the country and drug previously selected (**Fig. 2C**). Once a DRM is selected with a mouse click, three events are triggered: the DRM Selection title bar shows the selected DRM and drug, the atlases are color coded for the presence of the DRM in each country, and the DRM Observation by Country table is populated (**Fig. 2D**).

The DRM Selection table contains three columns with information about the DRM, type of DRM, and links to PubMed abstract(s) containing experimental evidence that supports the DRM and its classification (**Fig. 2C**). The first column in the table contains a color-coded list of the DRMs related to the selected drug. The IUPAC signal letter code for the wild-type amino acid, the amino acid position, and the identity of the mutated amino acid are shown (e.g. I47A) [12].

The type of DRM is displayed in the second column and each row is colored using a DRM classification scheme. GoMAP-HIV contains two different DRM classification schemes that can be chosen from a checkbox just below the DRM selection title bar. A subset of the data in the GoMAP-HIV database comes from the Stanford HIV Drug Resistance Database and had DRM classifications of major or minor [13]. GoMAP-HIV contains 188 DRMs from the Stanford Database that have one of these designations and selection of the checkbox below the title bar restricts the DRM selections to these DRMs.

As the default classification scheme, we provide a broader approach to classification. We implemented this new scheme because as we annotated DRMs from the literature and other databases, we observed DRMs that did not fit into the standard categories of major and minor [13]. Definitions for the new scheme can be found in **Table 1**. Briefly, DRM types designated *beneficial* or *beneficial set* (decrease drug resistance) are colored different shades of green. Those that cause resistance, *primary* and *primary set*, are colored red and pink, respectively. Those that amplify resistance are called *secondary set* and are colored purple. The few mutations that do not affect resistance directly, but which are precursors to other DRMs are called *precursors* and colored light blue. There is a checkbox option to view *ambiguous* mutations, which are colored white. Ambiguous mutations are those DRMs identified from another database where a published peer-reviewed primary source could not be identified.

**Table 1:** Definitions for different types of drug resistance mutations.

*Type of DRM*	*Definition*
Primary	Causes resistance without any other mutations
Primary set	Two or more mutations that cause resistance only in the presence of other primary set mutation(s)
Secondary	Enhances resistance caused by a primary mutation
Resistance precursor	A mutation that has no effect on resistance, but must occur prior to another primary or primary set of mutations
Beneficial	A mutation that prevents or reduces resistance
Beneficial set	Two or more mutations that when occurring simultaneously prevent or reduce resistance
Ambiguous	Cannot determine the nature based on existing literature

The DRM list is initially populated with all DRMs observed for the drug selected. However, using the mouse to click the “Observed only in selected Country” checkbox in the title bar will repopulate the DRM list showing only those DRMs observed in the country selected.

#### DRM Observation by Country window

Once a drug has been selected from the DRM Selection window, the title bar changes to include the DRM and drug (**Fig. 2D**). The first of three columns in this table contains an alphabetized list of all countries with DRMs related to the selected drug. Each row in the second and third columns indicates whether the DRM has been observed in naïve or treated patient blood samples from that country, respectively. In each column the numerator is the number of sequences in the country that have the DRM selected and the denominator is the total number of sequences in the country indicated. The information for the naïve- or treated-DRM is color-coded to match the atlas displays.

### Example GoMAP global analysis of Fosamprenavir DRMs

**Figure 3:**
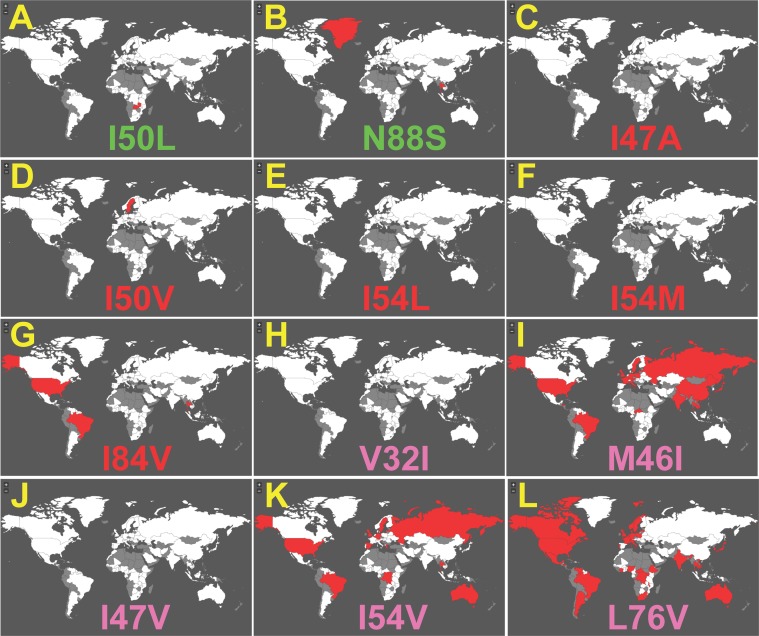
GoMAP example maps showing occurrence of DRMs for Fosamprenavir in genomes of naïve patients. Atlas displays for all 12 beneficial, primary, and primary set DRMs in the protease gene associated with Fosamprenavir and only for patient blood samples from drug-experienced patients (**A-L**). DRM type labels are colored as in the DRM table (**Fig. 2**).

**Figure 4:**
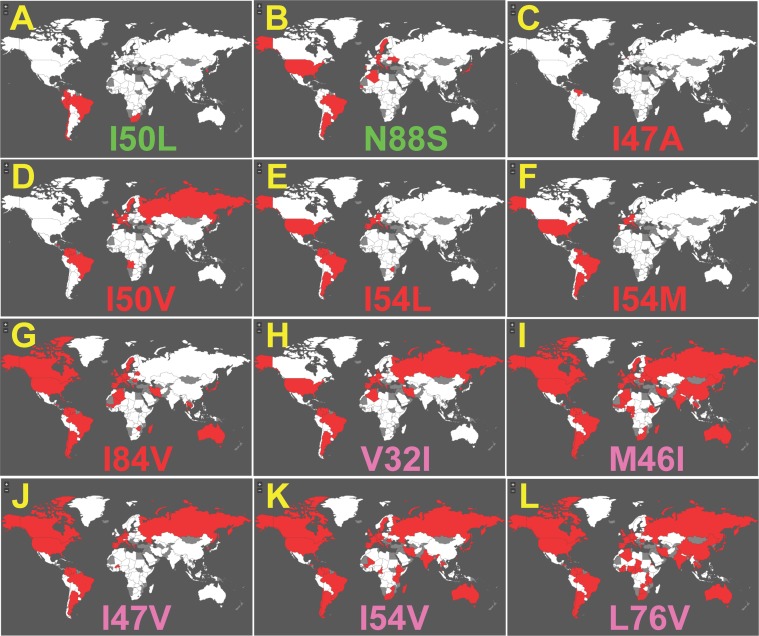
GoMAP example maps showing occurrence of DRMs for Fosamprenavir in genomes of drug experienced patients. Atlas displays for all 12 beneficial, primary, and primary set DRMs in the protease gene associated with Fosamprenavir and only for patient blood samples sequences from naïve patients (**A-L**). DRM type labels are colored as in **Fig. 3**.

GoMAP is the first tool that can be used to look at the distribution of individual HIV DRMs for HIV drugs. **Figs. 3** and **4** shows examples of geographic distribution plots generated for several DRMs in HIV protease previously associated with resistance to Fosamprenavir. For protease, there are 90,474 sequences from 120 countries. There are 16 different mutations in HIV protease that influence resistance to Fosamprenavir including: primary mutations I47A, I50V, I54L, I54M, I84V; primary set mutations V32I, M46I, I47V, I54V, and L76V, beneficial mutations I50L and N88S; and secondary set mutations L10F, L33F, M46L, and L90M [14]–[26].

The global distributions of individual DRMs for Fosamprenavir in naïve patients show interesting patterns (**Fig. 3**). As expected the 5 primary mutations were not ubiquitously observed, each showing up in only 0–3 countries. The two beneficial mutations had similar profiles. Of the 5 primary set mutations, 2 were not yet observed (V32I and I47V), but the other 3 were prevalent showing up in countries from most continents. In particular L76V was the most prevalent of the DRMs, observed in most countries of Europe, the Americas, and Australia.

In general, the global distribution of Fosamprenavir DRMs for patients previously receiving ARV therapy showed more wide spread prevalence (**Fig. 4**). Primary DRM I84V was the most widespread with occurrences in most countries in Europe, the Americas, and Australia. I47A has only been observed in Venezuela and other primary DRMs were observed in several countries in many continents. One major noticeable difference was for primary set mutations, with all showing generally widespread distributions on most continents.

## Discussion

The CDC, WHO and other agencies involved in managing the impact of pathogens on human health all have programs that focus on surveillance. These data, when mapped, assist in the investigation and management of outbreak events and epidemics. Indeed, surveillance studies have led to the identification of a growing concern in pathogen management. Since the landmark work documenting Methicillin-resistant *Staphylococcus aureas* (MRSA) in hospitals of Great Britain in the 1960s it has become clear that strains of pathogens can become resistant to antibiotics [27]. This has become more predominant with a number of bacterial and viral pathogens acquiring DRMs. Although there is a growing body of DRM data for a variety of pathogens, a tool that relates this data to geographic surveillance is missing. By combining the examination of geographic distribution of pathogen cases with the appearance of drug resistance mutations, we propose a new scheme that should aid in the management of pathogen-borne illness. Because this method relies on detecting drug resistance mutations in the genomes of pathogens and metadata for the geographic location of the sample, we call this new approach “Geogenomics”.

In order to demonstrate the feasibility and utility of Geogenomics, we sought to first examine a pathogen that had extensive data on genome sequences with geographical locations. HIV-1 met these criteria and we were able to obtain and use sequence-based data from the public Los Alamos HIV sequence database. We have built a Java-based web application called GoMAP. The GoMAP-HIV implementation presented herein focuses on HIV.

In addition to the sequence data, we needed a source of HIV-1 DRMs. We first attempted to use data sources from existing DRM databases, but noted that some DRMs reported in these databases were not supported by a published peer-reviewed science paper, a concept we have enforced in previous database/web systems we have built [28]–[30]. We therefore generated a consolidated HIV DRM database with records supported by at least one published paper (see methods). This annotation effort resulted in 1571 DRMs, 872 of which are for 23 known FDA-approved ARVs for treating HIV-1 infection.

23 different HIV ARVs can be investigated with GoMAP (Maraviroc is not included because GP120 is too polymorphic, nor is Dolutegravir due to its recent release to the market). We present a test case analysis of Fosamprenavir as an example of GoMAP-HIV (**Figs 3** **.** and **4**). We consistently observed the presence of primary DRMs in the drug-experienced patients from a small set of countries mostly clustered in central Europe. This geographic distribution is consistent with several studies previously reported. One study found that 29% of the French patients that were enrolled in the study and receiving Amprenavir/Ritonavir were drug resistant [31]. A second study found that 37% of the German patients enrolled in the study, who were previously treated with another protease inhibitor, were also resistant to Amprenavir [32]. Note that Fosamprenavir is a prodrug of Amprenavir and metabolized into Amprenavir in the intestine [33]. The example analysis of Fosamprenavir demonstrates the potential value in identifying countries where HIV sequences have DRMs that are known to be associated with resistance to specific drugs.

For GoMAP-HIV, we wanted to be as comprehensive as possible in our presentation of HIV DRMs. On the web system, we provide the standard major/minor classification scheme used by the IAS and Stanford HIV Drug Resistance database. However, we identified some limitations in this scheme. The designation “major” is defined as “mutations that reduce drug susceptibility by themselves”, and the term minor is used for “mutations that reduce drug susceptibility or improve the replicative fitness of isolates with a major mutation”[13]. In GoMAP, we have added additional DRM definitions to be inclusive of other types of mutations that we observed as we annotated the DRM data and set criteria for such DRM designations.

Major, occasionally called primary, is similar to what we are now calling *primary*. For primary DRMs in the GoMAP annotations, the mutation must be observed in a clinical patient blood sample that demonstrates significant drug resistance and when introduced into a clean viral background, produces significant resistance in culture without the presence of other mutations. We recognized that some DRMs only produce resistance when in combination, which does not fit well into the major and minor scheme. We call these *primary sets*.

Minor, occasionally called secondary, is similar to what we are now calling *secondary*. We also recognized the need for *secondary sets*, where sets of mutations are identified that enhance viral fitness when combined with a primary mutation. We also needed classifications that did not previously exist for *beneficial*, *beneficial set*, and *precursor* for mutations that did not fit into the major/minor classification scheme. This scheme is the default scheme used in GoMAP-HIV, but there is an option to use the Major/Minor scheme.

While GoMAP provides a system for examining a global view of HIV DRMs, we recognize that there are a number of limitations concerning the HIV data. One of the challenges with epidemiological studies is that there is a number of factors of concern in compiling data from different studies [34]. First, the sampling is biased. The different patient blood samples have been complied over a ∼30 year period. Each individual study has variable patient selection, assay methods employed, and interpretation by the investigators. Second, when an isolate is sequenced, we must consider that it represents one sequence of many circulating forms of the virus that contain mutations. Third, some countries, like Cambodia, have a low number of sequenced patient blood samples. Nevertheless, our implementation is intended to provide an example of a new approach that can be used for tracking and managing pathogens.

Another limitation is that some HIV DRMs were only observed upon treatment with two or more drugs at once and are not used in our plots because in this situation the ambiguity in the drug causing the DRM cannot be resolved. Another concern is that certain clades are known to have relatively specific geographical distributions and DRMs shown on GoMAP may somewhat represent clade distributions. Although most HIV-1 clades are thought to have similar sensitivity to drug resistance, there are specific cases where certain clades are more prone to drug resistance, e.g. [6], [35]. However, very few of our DRM maps show distributions that match previously published clade distributions [6].

We used GoMAP to mine for geogenomic DRM patterns. Although the data has limitations, it represents a rather large compendium of many studies with numerous sequences, and can provide some insight into the evolution of HIV and the geographic spread of particular DRMs.

We observed several patterns of regional concentration in DRMs that generally were not coincidental with the established global subtype distributions [36]. The I50L primary mutation in protease for Atazanavir and Amprenavir is predominant in South America, but not in the same countries as the B or BF subtype distributions. Likewise, the Y188H primary mutation in reverse transcriptase for Delavirdine and Nevirapine is in a cluster of 3 countries in central Africa in sequences for drug-experienced patients. The G190V primary mutation in reverse transcriptase for Nevirapine is in a small cluster of European countries. One obscure observation is that all 8 primary DRMs for Enfuvirtide are observed in Italy, and some of these primary DRMs are also in a few other counties; samples with viruses harboring some of these DRMs have been observed in Italian patients [37]. Collectively, the geographic patterns suggest a transmission-based spread for these DRMs.

The distribution of primary DRMs is of concern, because it could negatively impact ARV therapy, especially if the DRM is in naïve patients. Several primary DRMs had widespread global distributions. The I93L primary DRMs for Indinavir and L76V for Lopinavir are widespread, present in most countries, including naïve patients. Other primary DRMs with a global distribution are N89I, L90M for protease and E399D, V179I, and G196E for reverse transcriptase.

As expected, some DRMs are rarely observed in naïve patients, showing in only 0 or 1 country, but are widespread in patients that have undergone ARV therapy. These include V82T, V82S, L24I, and I47V for protease, and E44D, V106A, Y181V, H221Y and F227L for reverse transcriptase. This is typically observed in epidemiological studies, e.g. [38].

Surprisingly, a large number of published primary DRMs are not observed in any country for naïve or treated patients. These include DRMs (I47A, I84C) for protease, (P143S, K65N, K70E, K70G, M184T, Y181V, G190C, G190Q, F227C, K101P, K103T, K103H, I132M, Y181V, Y 188C, G190C, G190Q, E138R) for reverse transcriptase, and F121Y, Y143R, Y143C, and N155S for integrase. These mutations were observed in controlled experimentation; so why they have not appeared in this larger cohort of patients is unknown. One possibility is that these DRMs may provide resistance to ARVs in culture, but not so *in vivo*, perhaps due to differential recognition by the immune system.

The distribution of beneficial DRMs is of interest, because it could positively impact the efficacy of ARV therapy. We observed three beneficial mutations for Darunavir (K20I, E35D and V82A) that have a widespread global distribution, even in naïve patients. Another beneficial DRM with widespread distributions include A71V for Saquinavir. Notably the K70E beneficial DRM for Zidovudine was not observed in any country. However, the distribution of beneficial DRMs should be interpreted with caution because there are examples where a DRM is beneficial for one ARV, but a primary DRM for another ARV (e.g. N88S and L76V for protease).

The use of biogeography in tracking pathogens has recently emerged in several other laboratories with applications that are synergistic with GoMAP. Like the WHO and CDC, the Center for Disease, Dynamics, Economics and Policy also has a resistance map web system for bacterial pathogens that is synergistic with GoMAP (http://www.cddep.org/).

Another web tool, Healthmap advances surveillance mapping systems by integrating agency reporting, allowing rapid tracking of H1N1 Influenza outbreaks [39]. We considered enabling annual temporal tracking of HIV in GoMAP, since the Los Alamos HIV sequence database had meta data associated with the year that the sample was collected: unfortunately, the data were too sparse to generate useful maps. However, GoMAP can be adapted to include this type of feature. In a step toward mapping pathogen mutations, Supramap uses a similar concept for rapid tracking of pathogen spread but also includes genomics to identify and track movement of specific pathogen strains [40]. This can be used to examine how pathogens mutate over time and space. GoMAP is more specifically and synergistically focused on tracking the spread of drug resistance mutations for pathogens. Our implementation examining HIV provides a system and approach to examine HIV drug resistance mutations.

In conclusion, GoMAP embodies a new “Geogenomic” approach for examining pathogen mutations. We think this tool will be useful for examining the evolution of pathogens, examining how drug resistance spreads, and for those working in the area of public health. We offer our tool to public health agencies for their own use in tracking HIV mutations or mutations in other pathogens. GoMAP is an open access web application available at http://gomap.bio-toolkit.com/GoMap/. Documentation of GoMAP and a User Guide are at http://www.bio-toolkit.com/GoMAP/project. The source code and SQL database of DRMs for HIV is available upon request.

## Methods

### GoMAP-HIV implementation

GoMAP-HIV was implemented as a Java-based web application using standard three-tier software architecture consisting of a relational database for information management layer, a Java Servlet-based backend for data access, and a web-based presentation layer that is built using AJAX technology. The data was created by aligning amino acid sequences from patient blood samples for each country using ClustalΩ (http://www.clustal.org) and then analyzing the aligned sequences for individual DRMs. The presentation layer includes browser-based Geographical Information System (GIS) libraries (e.g. JVectorMap, http://www.jvectormap.com) and uses bioinformatics libraries to read and work with alignment files and the resulting aligned sequences (e.g. Strap, http://www.bioinformatics.org/strap/; BioJava, http://www.biojava.org). The JavaScript library jQuery (http://www.jquery.com) was also used in the presentation layer.
